# Event-related potential studies of outcome processing and feedback-guided learning

**DOI:** 10.3389/fnhum.2012.00304

**Published:** 2012-11-07

**Authors:** René San Martín

**Affiliations:** ^1^Department of Psychology and Neuroscience, Duke UniversityDurham, NC, USA; ^2^Center for Cognitive Neuroscience, Duke UniversityDurham, NC, USA; ^3^Facultad de Economía y Empresa, Centro de Neuroeconomía, Universidad Diego PortalesSantiago, Chile

**Keywords:** economics rewards, reward system, feedback-related negativity, reinforcement learning, dopamine, anterior cingulate cortex, P3, locus coeruleus-norepinephrine system

## Abstract

In order to control behavior in an adaptive manner the brain has to learn how some situations and actions predict positive or negative outcomes. During the last decade cognitive neuroscientists have shown that the brain is able to evaluate and learn from outcomes within a few hundred milliseconds of their occurrence. This research has been primarily focused on the feedback-related negativity (FRN) and the P3, two event-related potential (ERP) components that are elicited by outcomes. The FRN is a frontally distributed negative-polarity ERP component that typically reaches its maximal amplitude 250 ms after outcome presentation and tends to be larger for negative than for positive outcomes. The FRN has been associated with activity in the anterior cingulate cortex (ACC). The P3 (~300–600 ms) is a parietally distributed positive-polarity ERP component that tends to be larger for large magnitude than for small magnitude outcomes. The neural sources of the P3 are probably distributed over different regions of the cortex. This paper examines the theories that have been proposed to explain the functional role of these two ERP components during outcome processing. Special attention is paid to extant literature addressing how these ERP components are modulated by outcome valence (negative vs. positive), outcome magnitude (large vs. small), outcome probability (unlikely vs. likely), and behavioral adjustment. The literature offers few generalizable conclusions, but is beset with a number of inconsistencies across studies. This paper discusses the potential reasons for these inconsistencies and points out some challenges that probably will shape the field over the next decade.

## Introduction

The global function of the nervous system can be characterized as the adaptive control of behavior, a process that involves learning which action is relevant in a given context and switching to a different behavioral policy or scenario when outcomes are less optimal than expected. Hence “outcome” refers to the consequences that an organism faces as direct result of its own actions (e.g., financial losses due to impulsive investments) or resulting from its situation (e.g., receiving an unexpected gift). In order to successfully adapt behavior the brain has to determine, as quickly and as accurately as possible, whether the current scenario and behavioral policy results in positive or negative outcomes. This paper reviews the extant literature on how two event-related potential (ERP) components, the feedback-related negativity (FRN), and the P3, shed light on the neural substrate of outcome processing.

The relevance of outcome processing is highlighted by the association between individual differences in its functioning and personality constructs (Kramer et al., 2008; Onoda et al., 2010; Smillie et al., 2011), economic preferences (Coricelli et al., 2005; Kuhnen and Knutson, 2005; De Martino et al., 2006; Kable and Glimcher, 2007; Venkatraman et al., 2009), and pathological conditions such as compulsive gambling (Reuter et al., 2005; Goudriaan et al., 2006), drug abuse (Shiv et al., 2005; Everitt et al., 2007; Fein and Chang, 2008; Franken et al., 2010; Fridberg et al., 2010; Park et al., 2010), depression (Foti and Hajcak, 2009), and schizophrenia (Gold et al., 2008; Morris et al., 2008).

In the past decade neuroimaging studies have contributed enormously to identifying brain regions and patterns of functional connectivity supporting outcome processing in the human brain (e.g., Delgado et al., 2000, 2003; Breiter et al., 2001; Elliott et al., 2003; O'Doherty et al., 2003b; Ullsperger and Von Cramon, 2003; Holroyd et al., 2004b; Huettel et al., 2006; Kim et al., 2006; Mullette-Gillman et al., 2011). The discovery of this underlying physiology has been accompanied by a wealth of new knowledge about the functional properties of these mechanisms. In particular, non-invasive electrophysiological methods have provided important information about the temporal properties of the neural mechanisms mediating outcome evaluation in humans. Notably, by recording ERPs while participants perform learning-guided choice tasks or simple gambling games, researchers have begun to describe how the brain processes outcomes within a few hundred milliseconds from their onset.

**Figure 1:**
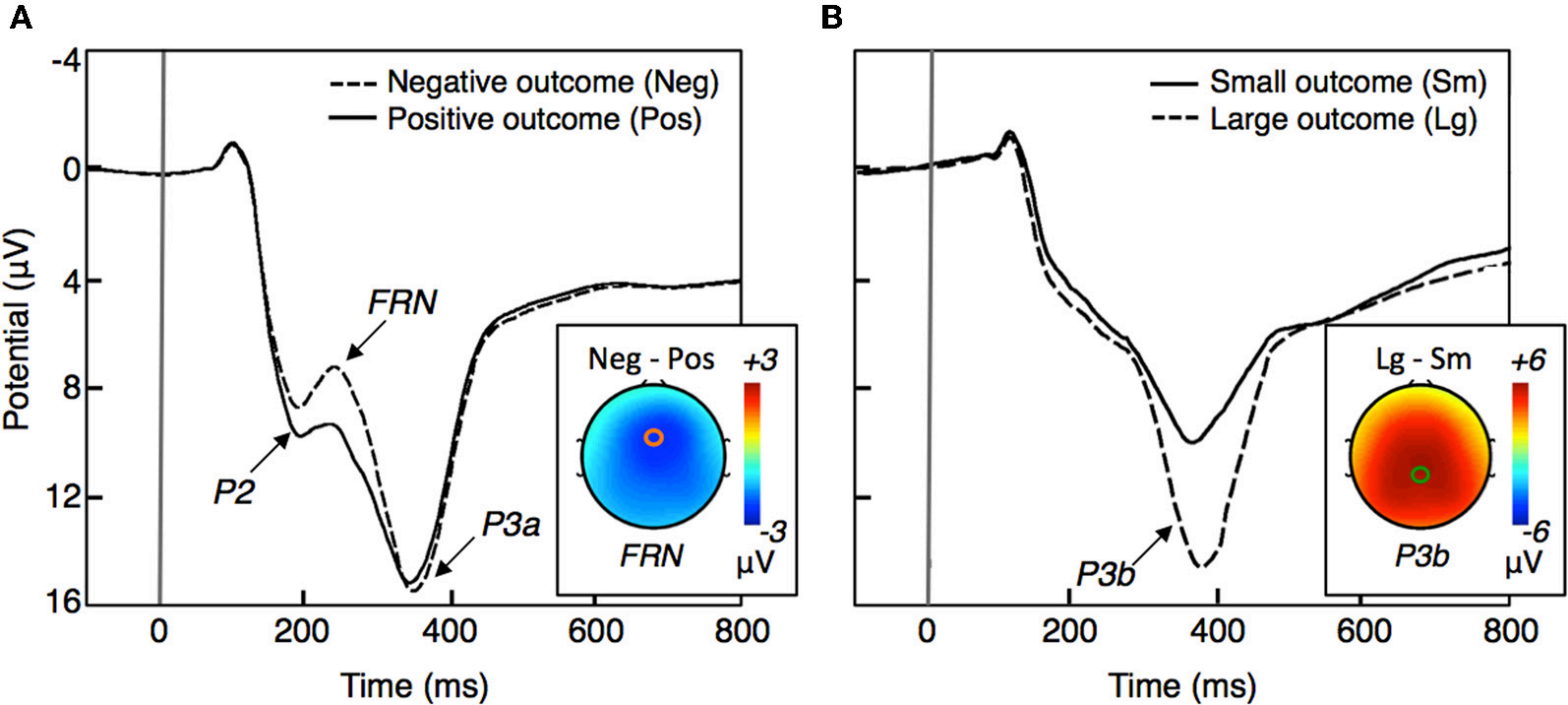
**A schematic representation of ERP waveforms typically elicited by outcomes (based on Gehring and Willoughby, 2002; Yeung and Sanfey, 2004; Goyer et al., 2008; Gu et al., 2011a).** The horizontal axis represents the elapsed time relative to the onset of behavioral feedback (at 0 ms). **(A)** Example of ERP waveform for negative and positive outcomes recorded at a frontocentral electrode site. The scalp topography represents the contrast between the two waveforms at the time when the FRN peaks (~250 ms). **(B)** Example of ERP waveform for large and small magnitude outcomes recorded at a central-parietal electrode site. The scalp topography represents the contrast between the two waveforms at the time when the P3b peaks (~300–600 ms).

Among the potential neural correlates of outcome evaluation, the FRN is by far the most studied ERP component. The FRN is a frontocentral negative-going ERP component that peaks ~250 ms following outcome presentation and is typically larger for negative outcomes than for positive outcomes (Figure 1A). According to source localization, the FRN is generated in the medial prefrontal cortex (mPFC), most probably in the anterior cingulate cortex (ACC; Miltner et al., 1997; Gehring and Willoughby, 2002; Ruchsow et al., 2002; van Schie et al., 2004; Muller et al., 2005; Nieuwenhuis et al., 2005b; Hewig et al., 2007; Yu and Zhou, 2009; Yu et al., 2011). Consistent with ERP studies, results implicating the ACC in processing negative feedback have been reported using fMRI (Kiehl et al., 2000; Holroyd et al., 2004b).

The P3 is another outcome-related ERP component. The P3 is a positive-polarity component most pronounced at the centroparietal recording sites at about 300–600ms after stimuli presentation (Figure 1B). According to a model proposed by Yeung and Sanfey (2004) outcome magnitude (i.e., large vs. small) and outcome valence (i.e., loss vs. gains) are coded separately in the brain, with the P3 being sensitive to outcome magnitude and the FRN to outcome valence. Despite controversial evidence (Hajcak et al., 2005, 2007; Bellebaum and Daum, 2008; Goyer et al., 2008; Wu and Zhou, 2009; Pfabigan et al., 2011) the independent coding model is presently the dominant account of the relationship between the FRN and the P3 during outcome processing.

The number of ERP studies of outcome have multiplied over the last decade. Yet the last systematic review of such studies focused exclusively on the FRN, and dates back 8 years (Nieuwenhuis et al., 2004a). This paper intends to provide an updated perspective about current knowledge from ERP research of outcome processing in the human brain. In order to achieve this goal, the paper reviews the historical antecedents and dominant theoretical accounts of the FRN and P3. These theories are evaluated in light of studies addressing how FRN and P3 are modulated by outcome valence (negative vs. positive), outcome magnitude (large vs. small), outcome probability (unlikely vs. likely), and behavioral adjustment. Finally, this paper discusses some challenges that ERP studies of outcome processing will probably have to address over the next decade in order to integrate FRN and P3 effects in a unitary account of outcome processing in the brain.

## The feedback-related negativity

### Historical antecedents of the FRN

The study of the neural basis of outcome evaluation and feedback-guided learning has been facilitated by the discovery of an ERP component, the FRN, which tends to distinguish between positive and negative outcomes. Miltner et al. (1997) was the first group to describe the FRN as an ERP component that is differentially sensitive to negative and positive feedback. In their study, they required participants to estimate the duration of a 1 s interval by pressing a button when they believed that 1 s had elapsed from the presentation of a cue. Their response was followed by the delivery of a feedback stimulus indicating whether their estimate was correct (positive feedback) or incorrect (negative feedback). A time window around 1 s was used to determine response accuracy and this window was adjusted so that the likelihood of positive and negative feedback stimuli for each participant were both 50%.

Miltner and colleagues found that the ERP elicited by negative feedback was characterized by a negative deflection at frontocentral recording sites with a peak latency of ~250 ms. This negativity was isolated with the method of difference waves, that is by subtracting the ERP response to positive feedback from the ERP response to negative feedback. Source localization estimates placed the generator of this difference wave near the ACC. The same results were found across different conditions in which feedback was provided in auditory, visual, and somatosensory modalities. Miltner and colleagues noted that the characteristics of this negativity corresponded in many respects (i.e., sensitivity to errors, polarity, scalp topography, and likely origin in the ACC) to those of the response-locked error-related negativity (ERN), an ERP component that reaches maximum amplitude about 100 ms following error commission in speeded response time tasks (Falkenstein et al., 1991; Gehring et al., 1993; Scheffers et al., 1996; for a review see Yeung et al., 2004). The authors suggested that both the ERN (elicited by error commission) and the FRN (elicited by negative feedback) reflect a general error detection function of the ACC. Indeed, converging lines of evidence from fMRI research (Kiehl et al., 2000; Holroyd et al., 2004b), magneto-encephalography (Miltner et al., 2003; Donamayor et al., 2011) and intracranial EEG recordings in humans (Wang et al., 2005) support the idea that the ACC is involved in performance monitoring and error detection.

### The reinforcement learning theory of the FRN

The error detection hypothesis (Miltner et al., 1997) was later extended by Holroyd and Coles (2002), who proposed that both the ERN and the FRN are scalp-recorded indexes of a neural system for reinforcement learning. This theory is based on research that implicates the basal ganglia and the midbrain dopamine (DA) system in reward prediction and reinforcement learning (Barto, 1995; Montague et al., 1996; Schultz et al., 1997; Schultz and Dickinson, 2000; Tobler et al., 2005; for a review see Schultz, 2002). From a computational standpoint, reinforcement learning problems involve a set of world states, a set of actions available to the agent in each state, a transition function which specifies the probability of moving from one state to another when performing a specific action, and a reward function, which indicates the reward or punishment associated with each transition (Ribas-Fernandes et al., 2011). In this context, the goal for learning is to discover, on a trial and error fashion, a policy (i.e., a stable mapping between states and actions) that maximizes the cumulative discounted long-term reward (Sutton and Barto, 1998).

According to the reinforcement learning theory of the ERN/FRN (RL-theory) the human brain solves reinforcement learning problems by implementing an “actor-critic architecture” (Barto, 1995; Joel et al., 2002). This theory assumes that several actors are implemented throughout the brain (e.g., amygdala, dorsolateral prefrontal cortex), each acting semi-independently and in parallel, and each trying to exert their influence over the motor system. According to the RL-theory, the ACC acts as a control filter, selecting a motor plan according to weighted state-action associations and communicating the corresponding response to the output layer (i.e., motor cortex) for execution.

The role of the critic in the actor-critic architecture is to evaluate ongoing events and predict whether future events will be favorable or unfavorable. When the critic revises its predictions for the better or for worse, it computes a temporal-difference reward prediction error (RPE). A positive or a negative RPE indicates that ongoing events are better than expected or worse than expected, respectively. The RPE is used to update both the value attached to the previous state and the strength of the state-action associations that determined the last response selection. The RL-theory attributes the role of the critic to the basal ganglia and assumes that the RPE signal corresponds to the phasic increase (for positive RPE, or +RPE) or decrease (for negative RPE, or −RPE) in the activity of midbrain DA neurons. Indeed, dopaminergic neurons in the monkey midbrain have been shown to code positive and negative errors in reward prediction (Schultz et al., 1997; Schultz and Dickinson, 2000; Tobler et al., 2005). According to the RL-theory, the RPE signal (i.e., phasic DA) is distributed to three parts of the network: (1) the adaptive critic itself (i.e., basal ganglia), where it is used to refine the ongoing predictions, (2) the motor controllers (e.g., amygdala), where it is used to adjust the state-action mappings, and (3) the control filter (i.e., ACC), where it is used to train the filter to select the most adaptive motor controller on a given situation. Together, the adaptive critic, the motor controllers, and the control filter learn how best to perform in reinforcement learning scenarios. The RL-theory proposes that the impact of DA signals on the ACC modulates the amplitude of the ERN/FRN (Holroyd and Coles, 2002). According to this theory, phasic decreases in DA activity enlarge ERN/FRN by indirectly disinhibiting the apical dendrites of motor neurons in the ACC, and phasic increases in DA activity reduce the ERN/FRN by indirectly inhibiting the apical dendrites of motor neurons in the ACC.

Despite a group of inconsistent findings that are reviewed in the next section (Hajcak et al., 2005; Oliveira et al., 2007; San Martin et al., 2010; Kreussel et al., 2012), the RL-theory remains as the dominant account of the FRN. Also, it has been an important factor contributing to the augment in the number of studies about this ERP component, mainly because of three reasons. First, the RL-theory speaks to one of the most explored issues in cognitive neuroscience, namely the cognitive functions implemented by the mPFC, and more specifically the ACC (Carter et al., 1998; Bush et al., 2000; Paus, 2001; Botvinick et al., 2004; Kerns et al., 2004; Ridderinkhof et al., 2004; Rushworth et al., 2004; Barber and Carter, 2005; Alexander and Brown, 2011). The RL-theory suggests that the general role of the ACC is the adaptive control of behavior, and that the ACC learns how to better perform its function from discrepancies between actual and optimal responses (for the ERN) or between actual and expected outcomes (for the FRN). This notion challenges the conflict-monitoring model (Botvinick et al., 2001; Botvinick, 2007) that characterizes the ACC as a region that can detect conflict between more than one response tendency, for example when a stimulus primes a pre-potent but incorrect response, or when the correct response is undetermined. While the conflict-monitoring model is consistent with neuroimaging evidence for ACC activation during error commission (Carter et al., 1998; Kerns et al., 2004) and with the characteristics of the ERN (Yeung et al., 2004), it does not address the FRN and is inconsistent with evidence from monkey neurophysiological studies that have not found any conflict-related activity in the ACC (Ito et al., 2003; Nakamura et al., 2005). The RL-theory, on the other hand, provides an explanation for both the ERN and the FRN, and accounts for ACC responses to outcomes in fMRI studies (Bush et al., 2002; Paulus et al., 2004), and in single-unit recording studies with monkeys (Niki and Watanabe, 1979; Amiez et al., 2005; Tsujimoto et al., 2006) and humans (Williams et al., 2004).

Second, the RL-theory proposes that the FRN reflects the evaluation of events along a general good–bad dimension, but it is non-specific about what actually constitutes a good or a bad outcome. Thus, the theory applies both to the difference between financial rewards and punishments (i.e., utilitarian feedback) and to the difference between correct trials and error trials (i.e., performance feedback). This factor makes the FRN especially relevant both for researchers interested in economic decision-making, and for researchers interested in the neural mechanisms of cognitive control. Gehring and Willoughby (2002) questioned the existence of such a highly generic mechanism by showing a medial frontal negativity (MFN) that was elicited by negative utilitarian feedback (loss of money vs. gain of money) but not by stimuli revealing that an alternative choice would have yielded a better result than the actual choice (i.e., performance feedback). However, in a subsequent study Nieuwenhuis et al. (2004b) showed that when feedback stimuli conveyed both utilitarian and performance information the FRN reflects either dimension of the information, depending on which aspect of the feedback is highlighted by the physical properties of the stimuli.

Finally, another contribution of RL-theory to the growing interest in the FRN is that this theory affirms that the events are treated as good or bad outcomes not in absolute terms but relative to their relationship with expectations. Since the FRN would reflect the difference between the actual and the expected outcomes, the FRN would potentially provide both a measure of the impact of events (i.e., experienced value) and a measure of anteceding expectations (i.e., expected value). Most of the research about outcome processing in the brain has explored these factors; several research groups have studied the modulation of the FRN by both properties of the outcome, such as valence (loss vs. win) and magnitude (large vs. small), or by properties of the context in which the outcome is presented, such as reward probability and the potential magnitude of reward. Few studies have also explored another critical component of the RL-theory, which implies that the amplitude of the FRN in a particular trial should predict the degree of behavioral adjustment in subsequent trials (Holroyd and Coles, 2002). The following section discusses the RL-theory in light of studies investigating the relationship between the FRN and outcome valence, outcome magnitude, outcome probability, and behavioral adjustment.

### Modulation of the FRN by outcomes and contexts

The RL-theory predicts that the amplitude of the FRN is correlated with the magnitude of the RPE. If this is true, negative outcomes should elicit larger FRNs than positive outcomes; larger negative outcomes should elicit larger FRNs than smaller negative outcomes; and unexpected negative outcomes should elicit larger FRNs than expected negative outcomes. The RL-theory also predicts an interaction between valence and magnitude and between valence and probability. For example, small gains should be associated with greater FRNs than large gains. Finally, according to the RL-theory the amplitude of the FRN on a given trial should predict behavioral adjustment on subsequent trials. This section examines the RL-theory in the context of studies addressing how the FRN is modulated by outcome valence (negative vs. positive), outcome magnitude (large vs. small), outcome probability (unlikely vs. likely), and behavioral adjustment.

#### Outcome valence

The most consistent finding in the FRN literature is that such ERP component is larger for negative feedback than for positive feedback. This valence dependency has been confirmed using monetary rewards (Gehring and Willoughby, 2002; Holroyd and Coles, 2002; Yeung and Sanfey, 2004; Goyer et al., 2008; Yu et al., 2011) and non-monetary performance feedback (Miltner et al., 1997; Luu et al., 2003; Frank et al., 2005; Luque et al., 2012). These studies have typically measured the FRN amplitude by computing the difference between the ERP elicited after negative outcomes and the ERP elicited after positive outcomes. This approach is most common because the overlap between the FRN and the P3 may distort the FRN net amplitude. However, a disadvantage to this method is that it does not inform whether the FRN corresponds to a negative deflection after negative outcomes or to a positive deflection after positive outcomes, or both. Indeed, a recent hypothesis argues that the differences between positive and negative outcomes could be better explained by a positivity associated with better than expected outcomes, rather than a negativity associated with worse than expected ones (Holroyd et al., 2008). This theory proposes that unexpected outcomes, regardless of their valence, elicit a negative deflection known as the N200 (Towey et al., 1980) and that trials with unexpected rewards elicit a feedback correct-related positivity (fCRP) that cancels the effect of the N200 component in the scalp-recorded ERP.

Recently, several groups have begun to quantify the FRN independently for positive and negative outcomes, and most of these studies have found greater modulation of the FRN to positive outcomes than to negative outcomes (Cohen et al., 2007; Eppinger et al., 2008, 2009; San Martin et al., 2010; Foti et al., 2011; Kreussel et al., 2012). However, at the same time these effects tend to disconfirm the fCRP-hypothesis, by showing a greater positivity for expected compared with unexpected gains (Oliveira et al., 2007; Wu and Zhou, 2009; San Martin et al., 2010; Chase et al., 2011; Yu et al., 2011; Kreussel et al., 2012).

In summary, the FRN is consistently larger for negative as compared to positive feedback, but it is not clear if this effect is due to a negative deflection following negative outcomes, a positive deflection following positive outcomes, both alternatives, or some unmentioned factor. This issue is critical for future research regarding the neurocognitive role of the FRN, and as of yet remains an open question.

#### Outcome magnitude

According to the RL-theory the FRN should not reflect a main effect of outcome magnitude given that the magnitude of an outcome can be evaluated as positive or negative only after considering the valence of such outcome (i.e., large gains are better than small gains, but the opposite is true for losses). Confirming this observation, several studies have noted the absence of a main effect of outcome magnitude (Mars et al., 2004; Yeung and Sanfey, 2004; Toyomaki and Murohashi, 2005; Polezzi et al., 2010). In a now-classic study, Yeung and Sanfey (2004) asked participants to select between cards that were unpredictably associated with monetary gains and losses of variable magnitude. The authors conclude in this study that the FRN was larger for losses compared with gains and did not show a main effect of outcome magnitude. However, others have reported that the FRN is larger for small magnitude outcomes, regardless of valence (Wu and Zhou, 2009; Gu et al., 2011a; Kreussel et al., 2012). This second set of studies did not elaborate on this finding, but rather as Wu and Zhou (2009) have suggested, the discrepancy could be related either to different approaches to measuring the FRN, or to the use of experimental paradigms where the expectancy toward reward magnitude was emphasized.

If the FRN does indeed code the difference between expected outcomes and obtained outcomes, it should be sensitive to deviations from expected reward magnitude (e.g., if the person expected a large loss, a small loss should be treated as a positive outcome). Aligned with this notion, Goyer et al. (2008) found that a model that includes both valence and magnitude explained a larger proportion of the variance associated with the FRN compared with a model that only includes valence. They also found that the difference between the FRN elicited by monetary losses and the FRN elicited by monetary gains was greater for large magnitude outcomes (i.e., −25¢ minus +25¢) than for small magnitude outcomes (i.e. −5¢ minus +5¢). In a related study, Holroyd et al. (2004a) found that the amplitude of the FRN depends on the range of possible outcomes in a given block. For instance, winning +2.5¢ elicited a smaller FRN when it was the best possible outcome than the same result when winning +5¢ was also possible. This FRN effect provided strong support to the RL-theory, according to which the FRN is an indirect measure of a firing pattern of DA neurons in the midbrain. Indeed, it has been shown that the same reinforcement can lead to phasic DA decreases if it is smaller than expected or phasic DA increases if it is larger than expected (Tobler et al., 2005).

Other studies have found effects that are less consistent with the RL-theory. Hajcak et al. (2006) reported that the FRN amplitude did not scale with the magnitude of the loss; Bellebaum et al. (2010) reported that the size of the potential reward affected the FRN amplitude in response to non-reward, but not to positive feedback; and San Martin et al. (2010) reported that the size of the potential reward affected the FRN amplitude in response to monetary gains, but not to monetary losses. These studies suggest that under some circumstances the FRN does not mirror a graded RPE. In this sense therefore, the existing literature is not entirely consistent with the RL-theory and new research is necessary to help resolve these disparate results.

#### Outcome probability

The probability of the experienced outcome is yet another factor that is crucial to the RPE and the RL-theory predicts that the probability of reward would affect the FRN responses to the upcoming outcome. Unexpected losses therefore should be associated with greater negativities than expected losses, and unexpected gains should elicit greater positivities than expected gains. Several studies have supported this prediction (Holroyd and Coles, 2002; Nieuwenhuis et al., 2002; Holroyd et al., 2003, 2009, 2011; Potts et al., 2006; Hajcak et al., 2007; Goyer et al., 2008; Walsh and Anderson, 2011; Luque et al., 2012). Nevertheless, with the exception of the study by Potts et al. (2006), these studies employed a difference wave approach (i.e., loss minus gain) and therefore it is not clear if the effects were driven by large negativities associated with unexpected negative outcomes or large positivities associated with unexpected positive outcomes, or some combination of both. Moreover, the difference wave approach might find an effect in the direction predicted by the RL-theory even if unexpected outcomes, regardless of valence, are associated with larger negativities, provided that the effect is greater for losses. A study by Oliveira et al. (2007) shed further light onto this issue. They found that positive feedback and negative feedback elicited a similarly large FRN when the actual feedback and the expected feedback mismatched. They reported a tendency for people to be overly optimistic about their own performance, and they found that because of this tendency there were three times more mismatches between expectancy and the actual feedback for erroneous trials than for correct trials. Similarly, other studies have reported that the FRN is elicited by unexpected outcomes, regardless of valence (Wu and Zhou, 2009; Chase et al., 2011; Yu et al., 2011).

Oliveira et al. (2007) proposed that the FRN reflects the response of the ACC to violations of expectancy in general (i.e., unsigned RPE), and not only for unexpected negative outcomes. Similar conclusions have been reached using fMRI in humans (Walton et al., 2004; Aarts et al., 2008; Metereau and Dreher, 2012) and single-unit recordings in monkeys (Niki and Watanabe, 1979; Akkal et al., 2002; Ito et al., 2003; Matsumoto and Hikosaka, 2009; Hayden et al., 2011). Also, a recent computational model (Alexander and Brown, 2011) has been able to simulate the FRN under the assumption that the mPFC is activated by surprising events, regardless of valence. However, some studies have reported effects that are hard to reconcile with Oliveira et al.'s account of the FRN. Both Potts et al. (2006) and Cohen et al. (2007) found that unexpected gains elicited larger positivities than expected gains, not larger negativities as Oliveira and colleagues suggest. In another study, Hajcak et al. (2005) reported that the FRN was equally large for expected and unexpected negative feedback; a result that is inconsistent with both Oliveira et al.'s account and with the RL-theory.

Broadly speaking, the evidence reviewed above does not permit conclusions about the modulation of the FRN by outcome probability. Both the RL-theory and the hypothesis proposed by Oliveira et al. (2007) have received empirical support, but these are mutually exclusive accounts. New research is needed in order to understand the relationship between FRN amplitude and outcome probability.

#### Behavioral adjustment

Convergent evidence has implicated the ACC in the flexible adjustment of behavior on the basis of changes in reward and punishment values. For example, a recent study showed that cingulate lesions in monkeys impaired the ability to use previous reinforcements to guide choice behavior (Kennerley et al., 2006). Another study, using a reward-based reversal learning paradigm, identified cells in the ACC of the monkey brain that fired only if reward was less than anticipated and if such reduction in reward was followed by changes in action selection (Shima and Tanji, 1998). In humans, fMRI studies of reversal learning have reported the same effect (Bush et al., 2002; O'Doherty et al., 2003a).

If neural RPE signals are used to guide decision-making and the FRN reflects the impact of such signals in the ACC, as suggested by the RL-theory, FRN magnitudes in response to decision outcomes should be related to adjustments in overt behavior. Evidence for this hypothesis was demonstrated by Luu et al. (2003) using a task in which participants had to respond to a target arrow with the hand indicated by the direction in which the arrow pointed. Luu and colleagues found that the amplitude of the FRN elicited by a feedback indicating that the response was slow correlated with subsequent speed of response. In another study, Frank et al. (2005) showed that the difference between the FRN elicited by negative and positive feedback correlated, across participants, with the difference between subjects' ability to learn to avoid negative feedback versus to learn to approach positive feedback. Similarly, Bellebaum and Daum (2008) found that violations of reward predictions modulated the FRN only in participants that were able to learn, through trial and error, and use a rule determining reward probability. However, the last two studies (Frank et al., 2005; Bellebaum and Daum, 2008) do not rule out the possibility that the reported effects are by-products of learning instead of underlying causes of individual differences in overt behavior.

Other researchers have found results that conflict with the RL-theory. For example, Mars et al. (2004) found that while subjects used the information provided by error feedback to adjust their behavior in a time judgment task, no relationship between FRN amplitude and behavioral adjustments was found. Specifically, more precise adjustments were evident following more versus less informative feedback, and larger behavioral adjustments were seen when subjects received feedback that implied a large error than following feedback suggesting a small error. However, FRN amplitude was smaller in the informative conditions, and was not influenced by the degree of error. In another study, Walsh and Anderson (2011) presented a decision-making task with two conditions: a no instruction condition in which participants received feedback about whether their choices were rewarded and had to learn reward probabilities by trial and error, and an instruction condition, where they additionally received a description of the association between cues and reward probabilities before performing the task. The authors found that instruction eliminated the association between feedback and behavioral adjustment, but the FRN still changed with experience in the instruction condition. These studies suggest that, at least under some circumstances, the FRN can be elicited in the absence of behavioral adjustment, and behavior can be adjusted in the absence of a concomitant FRN effect.

Other studies do report a relationship between the FRN and behavioral adjustment, but not in the direction that the RL-theory predicts. For example, Yeung and Sanfey (2004) reported that after a large loss of money participants tended to repeat the selection of the larger magnitude (i.e., risky option), particularly if the preceding outcome elicited a large FRN. Similarly, using a computer Blackjack gambling task, Hewig et al. (2007) found that those participants exhibiting increased FRNs when losing after making risky choices showed a strong tendency to switch to even more risky choices. These results represent a challenge for the RL-theory, which predicts that participants should be less likely rather than more likely to perseverate in a response strategy after a large FRN.

One of the strengths of the RL-theory is that it provides predictions at the trial-by-trial level. Computational models can be fitted to behavioral data and the FRN can be compared with values derived from such models, such as the trial-by-trial fluctuation of the RPE during the task. Two studies found dissimilar results using that approach. In one study, participants played a competitive game called “matching pennies” against a simulated opponent (Cohen and Ranganath, 2007). On each trial, the subject and the computer opponent each selected one of two targets. If the subject and the computer opponent chose the same target, the subject lost one point, and if they chose opposite targets, the subject won one point. Supporting the RL-theory, the authors found that the FRN elicited by losses was more negative when subjects chose the opposite versus the same target on the subsequent trial. In another study, Chase et al. (2011) reported that the FRN amplitude was positively correlated with the magnitude of the negative RPE (−RPE), but negative outcomes that preceded behavioral adjustments were not accompanied by enlarged FRNs. The discrepancy between the results found by these studies could be explained by the characteristics of their experimental paradigms. The task used by Cohen and Ranganath (2007) discouraged the adoption of explicit rules or strategies. The simulated opponent was preprogrammed to choose randomly unless it was possible to find and exploit patterns in the behavior of the human participant. In contrast, in the study by Chase et al. (2011) participants were instructed to switch choice behavior only when they were sure that a rule determining the stimuli-response mapping had changed, and not after each exception to that rule. Indeed, in the study by Chase and colleagues the first violations of the rules were associated with greater −RPEs and greater FRNs, but not with behavioral adjustment.

In summary, different studies suggest that, at least in some learning situations, the processes underlying the generation of the FRN might be dissociated from the processes responsible for behavioral adjustments. It is possible, like the results from Chase et al. (2011) and Walsh and Anderson (2011) suggest, that the RL-theory does not account for behavioral adjustment under some learning context, but still predicts trial-by-trial fluctuations in FRN amplitude.

## The P3

### Historical antecedents of the P3

The P3 is a positive large-amplitude ERP component with a broad, midline scalp distribution, and with peak latency between 300 and 600 ms following presentation of stimuli. First reported in 1965 (Desmedt et al., 1965; Sutton et al., 1965), the P3 is perhaps the single most studied component of the ERP, probably because it is elicited in many cognitive tasks involving any sensory modality. The antecedent conditions of the P3 have been extensively explored using the so-called “oddball” paradigm (Duncan-Johnson and Donchin, 1977; Pritchard, 1981; Murphy and Segalowitz, 2004; Campanella et al., 2012), in which low-frequency target stimuli (*oddballs*) are embedded in a train of non-target stimuli (*standards*). Typically the subject is required to actively respond to each target stimulus. Using this task, Duncan-Johnson and Donchin (1977) were the first to report a correlation between the probability of an eliciting stimulus and the P3 amplitude. Specifically, they found that P3 amplitude was inversely proportional to the frequency or probability of the target stimuli in an oddball sequence (for an extensive review, see Nieuwenhuis et al., 2005a).

### The context updating hypothesis and the neural sources of the P3

The most influential account of the P3 is the context updating hypothesis (Donchin, 1981; Donchin and Coles, 1988). In this framework, the P3 indexes brain activity underlying the stimuli-induced revision of a mental model of the task at hand. If subsequent stimuli deliver information that mismatches with part of such model, the model is updated, with the amplitude of the P3 being proportional to the amount of cognitive resources employed during the revision the model.

There is an important degree of uncertainty regarding the neural generators of the P3. Lutzenberger et al. (1987) argued that large-amplitude potentials like the P3 must have widespread sources. Consistent with this view, intracranial P3-like activity has been recorded from multiple cortical areas (for a review see Soltani and Knight, 2000). Probably the most likely neural sources of the P3b can be found in a region that includes the temporal-parietal junction (TPJ; consisting of the supramarginal gyrus and caudal parts of the superior temporal gyrus) and adjacent areas (Kiss et al., 1989; Smith et al., 1990; Halgren et al., 1995). Indeed, studies have shown that lesions of the TPJ region produce marked reductions of the P3 associated with infrequent, task-relevant stimuli (Yamaguchi and Knight, 1992; Verleger et al., 1994; Knight and Scabini, 1998).

P3-like potentials have also been observed in medial temporal lobe (MTL) structures, including hippocampus and amygdala in cats (Kaga et al., 1992), monkeys (Paller et al., 1992), and humans (Halgren et al., 1980; McCarthy et al., 1989; Smith et al., 1990). The thalamus is another deep structure that produces P3-like potentials in humans (Yingling and Hosobuchi, 1984). However, biophysical considerations indicate that the possible contributions of deep cortical structures like the MTL and thalamus to the scalp-recorded electroencephalogram (EEG) are much too small (Lutzenberger et al., 1987; Birbaumer et al., 1990) and thus, the available evidence suggests that TPJ and adjacent areas are the most likely neural sources of the scalp-recorded P3.

### The locus coeruleus–P3 hypothesis

A more recent hypothesis proposes that the P3 reflects the neuromodulatory effect of the locus coeruleus (LC) norepinephrine (NE) system in the neocortex (Nieuwenhuis et al., 2005a). This hypothesis was the first account of the P3 based on neuroscientific knowledge and it is supported both by similarities between the target areas of NE projections and likely P3 generators, and by similarities between the antecedent conditions for phasic increases in NE and the antecedent conditions for P3 generation. The main claim of the “LC-P3 hypothesis” is that the P3 reflects a LC-mediated enhancement of neural responsivity in the cortex to task-relevant stimuli. Indeed, it has been shown that NE increases the responsivity of target neurons, and that such enhanced gain produces an increase in the signal-to noise ratio of subsequent processing (Servan-Schreiber et al., 1990).

The main source of NE for the forebrain is provided by the LC which is a small nucleus in the pontine region of the brain stem. Within the neocortex, NE innervation is particularly high in the prefrontal cortex and parietal cortex (Levitt et al., 1984; Morrison and Foote, 1986; Foote and Morrison, 1987). Dense NE innervation for the thalamus, amygdala, and hippocampus has also been reported (Morrison and Foote, 1986). Building on this overlap between NE targets and likely P3 generations is one of the main strengths of the LC-P3 hypothesis. Also, the latency of the LC phasic response (150–200 ms post-stimulus), added to the time course of NE physiological effects (100–200 ms post-discharge) (Aston-Jones et al., 1980; Foote et al., 1983; Pineda, 1995; Berridge and Waterhouse, 2003), is consistent with the typical P3 latency.

Phasic activity of the LC-NE system is sensitive to various aspects of stimuli to which the P3 amplitude is also sensitive, including motivational significance, probability of occurrence, and attention allocation. Similarly to the P3, LC phasic activity is generally more closely related to the arousing nature of a given stimulus than to the affective valence of the stimulus (Berridge and Waterhouse, 2003). For example, phasic LC responses occur following both positive and negative outcomes, provided that such outcomes require the animals to update their model of the environment (Rasmussen et al., 1986).

Noting the similarities between the context updating hypothesis and the involvement of the LC-NE system during learning, Nieuwenhuis (2011) reinterpreted the LC-P3 hypothesis and the context updating hypothesis as being complementary rather than competing accounts of the P3; with the LC-P3 hypothesis providing a mechanistic explanation, grounded on neuroscientific evidence, to the more abstract context updating hypothesis. In this sense, an important contribution of the LC-P3 theory is to connect studies of the P3 with new accounts about the role of the NE-mediated attention during learning. For example, it has been proposed that phasic NE is a generic signal indicating the need to attend to the environment and learn from it (Bouret and Sara, 2004), or that phasic NE encodes unexpected uncertainty (i.e., surprise in supposedly non-volatile environments) about the current state within a task, and serves to interrupt the ongoing processing associated with the default task state (Yu and Dayan, 2005; Dayan and Yu, 2006). By conceptually linking the P3 with theories about the role of NE-mediated attention during learning, the LC-P3 hypothesis provides an initial framework to interpret the functional role of the P3 in tasks involving outcome evaluation.

### Modulation of the P3 by outcomes and contexts

Applied to outcome evaluation and learning, the LC-P3 hypothesis predicts that outcomes associated with high levels of arousal or task-relevance (e.g., indicating the need for behavioral adjustment) will be associated with a large P3. The specific conditions associated with these factors, however, may change depending on the goal and the context of the task at hand. For example, losing when losses could be avoided should elicit a larger P3 than losing when losses are unavoidable. This section evaluates whether the LC-P3 hypothesis provides a plausible account of the P3 in brain studies of outcome evaluation and feedback-guided learning.

#### Outcome valence

Early ERP studies of outcome processing suggested that feedback indicating a bad performance (i.e., negative feedback) elicited larger P3s than positive feedback (Squires et al., 1973; Picton et al., 1976). However, subsequent studies showed that, when equated for probability of occurrence, positive and negative feedback elicited equally large P3s (Campbell et al., 1979), and that large P3s were elicited both by negative outcomes when participants thought they made a correct response, and by positive feedback when participants thought they made an incorrect response (Horst et al., 1980). These studies suggested that the effect of valence might have artificially emerged from the well-known sensitivity of the P3 to stimulus probability (see results with the oddball paradigm on section “Historical Antecedents of the P3”).

Using monetary rewards, Yeung and Sanfey (2004) found that the P3 was sensitive to reward magnitude but insensitive to reward valence. This result seems to fit with the idea that a large P3 is observed both to affectively negative and positive stimuli, provided that they are matched according to subjective ratings of arousal (Johnston et al., 1986; Keil et al., 2002). The claim that the P3 is insensitive to outcome valence has been supported by some studies (Sato et al., 2005; Yeung et al., 2005; Gu et al., 2011b), but there has been more evidence implicating that outcome valence does in fact modulate the P3. Some studies have reported that losses elicit larger P3s than gains (Frank et al., 2005; Cohen et al., 2007; Hewig et al., 2007), but surprisingly most of the studies have reported the opposite pattern, with larger P3s for gains than for losses (Toyomaki and Murohashi, 2005; Hajcak et al., 2007; Bellebaum and Daum, 2008; Wu and Zhou, 2009; Bellebaum et al., 2010; Polezzi et al., 2010; Zhou et al., 2010; Gu et al., 2011a; Kreussel et al., 2012). Importantly, in all of the studies reporting larger P3s after losses than after gains, participants could actually learn to avoid losses, so that the probability of losing decreased with practice. It is, therefore, possible that the effect of valence was artificially derived from the P3 sensitivity to stimulus probability. However, two studies reporting larger P3s for gains than for losses share the same characteristic (Bellebaum and Daum, 2008; Bellebaum et al., 2010). Moreover, Bellebaum and Daum (2008) found larger P3s for gains than for losses even when gains were more likely than losses. New studies might try to determine under what conditions the P3 is insensitive to outcome valence, and under what conditions it is increased for gains or increased for losses.

Recent studies have reported effects of the interaction between outcome valence and other factors. Wu and Zhou (2009) found that the difference between the P3 elicited by gains and losses (larger P3s for gains in this case) was eliminated when the amount of reward was inconsistent with the expectation built upon a preceding cue. Following the idea that the P3 might reflect the amount of cognitive resources allocated for stimulus processing (Donchin and Coles, 1988), the authors suggested that the inconsistency between the actual and the expected outcome magnitude might capture a large amount of attentional resources, such that the attention allocated to process outcome valence is reduced. A goal for new studies might be to replicate this effect and to specify under what circumstances the brain might need to distribute attentional resources between outcome variables.

In another study, Zhou et al. (2010) reported the same main effect of outcome valence, but in this case the difference was enlarged by action choice as compared to inaction. The authors suggest that action might increase the affective significance of gains. However, it is not clear why this would not also be true for losses.

In summary, the current evidence seems to disconfirm the hypothesis that the P3 is insensitive to outcome valence. Nevertheless, it is not clear whether the P3 is larger for gains or larger for losses. New studies are needed in order to clarify this issue. Also, and as already commented, early studies (Campbell et al., 1979; Horst et al., 1980) suggested that the effect of valence might result from the effect of another, more primitive, variable such as stimulus probability or affective involvement during the tasks. Contemporaneous studies need to take this possibility into consideration, during the experimental design, data analysis, and when forming conclusions.

#### Outcome magnitude

The independent coding model (Yeung and Sanfey, 2004) claims that the P3 is insensitive to valence and sensitive to outcome magnitude. Although the evidence reviewed in the previous section poses doubts on the first claim, the second claim is widely supported by the literature. Most of the studies that have tested the main effect of outcome magnitude have found more positive P3 responses to large magnitude outcomes than to small magnitude outcomes (Toyomaki and Murohashi, 2005; Goyer et al., 2008; Wu and Zhou, 2009; Bellebaum et al., 2010; Polezzi et al., 2010; Gu et al., 2011a; Kreussel et al., 2012). In order to interpret this effect, these studies have typically appealed to the concept of “motivational significance,” or the relevance of a stimulus for the current task (Duncan-Johnson and Donchin, 1977).

Motivationally significant or relevant stimuli are presumed to capture a large amount of attentional resources, and the P3 amplitude is supposed to scale with those attentional resources (Donchin and Coles, 1988; Nieuwenhuis et al., 2005a). Under this interpretation, large magnitude outcomes might receive more attention and have a greater impact on memory than small magnitude outcomes because they are more relevant for the final outcome of the session (cf. Adcock et al., 2006). Indeed, in the context of monetarily rewarded tasks, large magnitude outcomes have a greater impact in cumulative earnings, for better or for worse, depending on the outcome valence.

Recent studies have reported other effects involving outcome magnitude. Bellebaum et al. (2010) reported that not only the magnitude of the actual reward, but also the potential reward magnitude, modulated the P3. They used a task in which, on each trial, subjects had to guess the location of a coin that was hidden in one of six boxes. At the beginning of each trial, subjects were informed about the amount of money that they could win (i.e., 5¢, 20¢, or 50¢). Interestingly, the P3 elicited by non-rewarding outcomes (i.e., 0¢) scaled with the magnitude of the informed potential reward. In another study, Wu and Zhou (2009) found that the effect of outcome magnitude (i.e., larger P3s for large rewards than for small rewards) was eliminated when the reward amount was inconsistent with the expectation built upon a preceding cue. As already mentioned in the previous section, Wu and Zhou suggested that all attentional resources might be allocated by such inconsistency with expectations, leaving no resources available to process other outcome-related variables.

In summary, the P3 is consistently modulated by outcome magnitude, being more positive for large magnitude outcomes than for small magnitude outcomes. This effect may reflect that the motivational significance of outcomes scales with outcome magnitude. According to this interpretation both the actual and the expected magnitude of rewards determine the motivational significance of the outcome.

#### Outcome probability

Studies employing the classic oddball paradigm and manipulating the probability of the appearance of a particular stimulus showed that the P3 is more positive for infrequent stimuli than for frequent stimuli (Courchesne et al., 1977; Duncan-Johnson and Donchin, 1977; Johnson and Donchin, 1980). In the context of learning tasks, an early study reported that the largest P3s were elicited by negative feedback when participants thought they made a correct response, and by positive feedback when participants thought they made an incorrect response (Horst et al., 1980). Given this evidence, it has long been recognized that the P3 is modulated by stimulus probability, with more positive amplitudes elicited by unlikely our unexpected stimuli than to likely or expected stimuli.

Studies using monetary rewards and manipulating reward probability have widely supported the conclusion that the P3 is larger for unexpected outcomes than for expected outcomes, regardless of valence (Hajcak et al., 2005, 2007; Bellebaum and Daum, 2008; Wu and Zhou, 2009; Xu et al., 2011). These results are consistent with the context updating hypothesis stating that unexpected outcomes signal the need to update a mental model and that the P3 reflects the amount of cognitive resources allocated to this updating process. The results are also consistent with the LC-P3 hypothesis stating that the P3 reflects the impact of phasic NE in the neocortex. Indeed, it has been noted that increasing stimuli probability reduces the magnitude of phasic LC responses (Alexinsky et al., 1990; Aston-Jones et al., 1994), and phasic LC responses have been proposed to code unexpected uncertainty or surprise (Yu and Dayan, 2005; Dayan and Yu, 2006).

Two studies, however, have reported results that pose doubts into the modulatory effect of probability over the P3. While the P3s following wins were significantly affected by probability in these two studies, with unlikely wins eliciting larger P3s than likely wins, P3s for losses were either not modulated by probability (Cohen et al., 2007) or modulated in the opposite direction (Kreussel et al., 2012) (i.e., larger P3s for expected losses than for unexpected losses). After observing that the probability effect was maximal over anterior sites, Cohen et al. (2007), suggested that their results were more related with the FRN than with the P3. Indeed, and given that the FRN for unexpected losses tend to be larger (i.e., more negative) than the FRN for expected losses, the overlap between the FRN and the P3 may also explain the effect reported by Kreussel et al. (2012). Disentangling different outcome-related ERP components is one of the main challenges for ERP studies of outcome processing.

In summary, a large amount of evidence, coming both from classical studies using the oddball paradigm and from more recent studies using learning and gambling tasks, support the idea that the P3 is larger for unexpected events than for expected events, regardless of the event valence. Some studies have reported results contradicting this claim, but methodological considerations related to the overlap between the P3 and the FRN appear to provide a plausible explanation for such discrepancy.

#### Behavioral adjustment

Learning-guided decision-making tasks typically require storing and dynamically adjusting information about state-choice-outcome contingencies. Convergent evidence suggests that the LC-NE system contributes to learning this type of association. For example, it has been noted that the injection of a drug that increased the firing of LC neurons in rats promotes the animal's adaptation to changes in the behavioral requirements of a reinforcement-learning task (Devauges and Sara, 1990). In the monkey, LC activation has been reported to be restricted to task-relevant stimuli that require a behavioral shift (Aston-Jones et al., 1999). Building on this evidence, Bouret and Sara (2005) proposed that phasic NE could provoke or facilitate the dynamic reorganization of the neural networks determining the behavioral output. Similarly, Dayan and Yu (2006) proposed that NE signals encode unexpected surprise, serving to interrupt the ongoing processing and concentrate attentional resources in behavioral adjustment.

If, as the LC-P3 hypothesis proposes, the P3 reflects the NE-mediated enhancement of signal transmission in the cortex during the stimulus-induced revision of an internal model of the environment, a large P3 at the time of outcome processing should predict a large behavioral adjustment. In one of the few studies that have explored the relationship between the P3 and behavioral adjustment, Yeung and Sanfey (2004) found that individual differences in the P3 elicited by alternative, unchosen outcomes were related to behavioral adjustments. After dividing the participants into two groups on the basis of the size of their behavioral adjustment after trials in which they failed to select a card associated with a large win, they found that the difference in the P3 amplitude elicited by large-gain and large-loss alternative outcomes was larger in the participants showing a greater behavioral adjustment. In another study, Chase et al. (2011) found that P3 amplitude was greater for negative outcomes that preceded behavioral adjustment than for negative outcomes that did not precede behavioral adjustment in a probabilistic reversal-learning paradigm.

Evidence that dissociates the P3 from behavioral adjustment has also been reported. Specifically, Frank et al. (2005) found that, across participants, the difference between the ability to learn to avoid losses and the ability to learn to approach gains was predicted by the difference in the FRN amplitude elicited by negative and positive feedback, but not by the P3. Interestingly, the results found by Chase et al. (2011) showed the opposite pattern, with the P3 but not the FRN predicting behavioral adjustment. A possible reason for the discrepancy is that in Frank et al.'s study the need for adjustment was always signaled by losses, and as already reviewed the most consistent finding about the FRN is that it is larger for losses than for gains. In contrast, in the study by Chase and colleagues participants were explicitly instructed to switch choice behavior only when they were sure that a rule determining the stimulus-outcome contingencies had changed, and not after losing *per se*. Another possibility for this discrepancy is that both the FRN and the P3 code behavioral adjustment, but their relative involvement in this process depends on the goal of the task at hand or on the level of information processing that is required to adjust behavior. Evidence is scant to strongly support any of these possibilities, and future research is needed to determine the factors determining the relative involvement of the FRN and the P3 in behavioral adjustment.

## Discussion

The studies reviewed here suggest that the brain mechanism underlying the FRN and the P3 are consistently involved in outcome processing, but at the same time the literature shows an important degree of scientific uncertainty regarding the factors that affect the amplitude of these ERP components. Although the studies reviewed here have enough trials per condition (Marco-Pallares et al., 2011) and sample sizes that allowed them to detect significant results, reaching conclusions that generalize across studies have proven to be difficult. The FRN tends to increase its amplitude in response to negative outcomes and the P3 tends to increase its amplitude in response to arousing or task-relevant outcomes, but there are important exceptions in the literature that weaken the generalization of these statements.

In order to advance a model that integrates FRN and P3 effects in a unitary and real-time account of outcome processing, ERP studies of outcome processing will have to address methodological, empirical, and conceptual challenges in the upcoming years. First, optimizing paradigm design will be critical for being able to generalize conclusions about the role of the FRN and the P3 during outcome processing. Second, in order to allow a reliable comparison between studies, the field will have to advance toward standard methods to measure the ERP components. Third, ERP studies have intrinsic limitations for identifying brain regions and networks involved in outcome processing. Complementary techniques should be increasingly used to overcome such limitations. Fourth, studies interested in the effect of outcome variables on the FRN and P3 have typically involved different task demands (e.g., passive observation of outcome, active decision-making, etc.). Determining the impact of task demands on these ERP components will be crucial to be able to generalize conclusions about their role. Finally, the studies reviewed here portray outcome processing as two relatively disconnected processes. Our modern view of the brain, however, suggests that outcome processing probably involves several subprocesses concurring and interacting in time. ERP studies should take advantage of their high temporal resolution to study the temporal cascade of outcome processing in the brain. These challenges are further discussed in the remainder of the article.

### Methodological challenge: optimizing paradigm designs

During the last decade, ERP studies of outcome processing have produced a wealth of evidence employing a rich variety of experimental paradigms. While this heterogeneity is needed to generalize conclusions about the properties of a neural correlate beyond a particular experimental task, there is a risk associated with designing tasks that do not adequately isolate the variables of interest. For example, as already commented, early P3 studies concluded that negative feedback elicited larger P3s than positive feedback, but subsequently Campbell et al. (1979) showed that these results mostly reflected the well-known effect of stimulus probability on the P3.

More contemporaneous studies, especially some of those using feedback-guided learning, could be associated with a similar confound. These groups found that losing was associated with larger P3s than winning, but it is equally possible that this result reflects a probability effect: as learning progresses, losing becomes less likely. In order to dissociate a valence effect from a learning/probability/expectancy effect, ERP studies of outcome processing might benefit from measuring ERP components on different stages of the experimental session, or by comparing the ERP response from participants that demonstrate learning with those who do not.

Another confound that can limit the validity of the results is the potential gap between the goal that the participants really pursue during an experimental session and the goal that the experimenter is trying to elicit. For example, paradigms designed for studying the modulatory effect of participants' prediction on brain activity elicited by gains and losses might unintentionally emphasize the goal of predicting the upcoming outcome. If participants are asked about their belief on the incoming outcome, they might even perceive a predicted loss as a positive feedback (i.e., a correct prediction). To limit the effect of this confound while still being able study the effect of participants' predictions, paradigms should emphasize the goal of ensuring gains and avoiding losses. For example, experimenters could make outcomes contingent on participants' behavior and not purely probabilistic. Also, researchers could benefit from verbal reports about the task and its goals during pilot studies.

The study of outcome processing is associated with a large number of variables (i.e., outcome valence, outcome magnitude, expectancy toward magnitude, probability of winning, learning, motivation, etc.) that, depending on the task at hand, might covary in a way that undermines empirical results. Paradigms should be designed acknowledging this complexity in a way that minimizes the chances of introducing confounds.

### Methodological challenge: advancing toward standard measurement methods

An inherent difficulty of the ERP technique is that, because of potential component overlap, the comparison of ERPs elicited by different experimental conditions is often difficult to interpret (Luck, 2005). Although this problem is inherently technical, the manner by which it is addressed in each study may determine empirical results and functional interpretations. Research focused on the FRN has traditionally tried to solve this issue by creating difference waves (e.g., loss minus win) (Miltner et al., 1997; Holroyd and Coles, 2002; Nieuwenhuis et al., 2002; Mars et al., 2004; Hajcak et al., 2005, 2007; Potts et al., 2006; Holroyd et al., 2009, 2011; Walsh and Anderson, 2011; Xu et al., 2011). The problem with this approach is that it does not resolve the question whether the FRN corresponds to a negative deflection in one condition or to a positive deflection in the other condition. More recent studies have highlighted the importance of this issue by showing that, when gains and losses are measured separately, even the definition of the FRN as negative deflection that distinguishes negative from positive outcomes can be questioned (Oliveira et al., 2007).

Two alternative methods to measure ERP components have been systematically employed in ERP studies of outcome processing. The first approach is to compute the mean amplitude in a time window defined for each ERP component (e.g., 200–300 ms for the FRN) post-onset of the outcome and to enter that mean amplitudes into statistical analyses (Gehring and Willoughby, 2002; Ruchsow et al., 2002; Nieuwenhuis et al., 2004b; Yeung et al., 2005; Cohen et al., 2007; Cohen and Ranganath, 2007; Hewig et al., 2007; Bellebaum and Daum, 2008; Goyer et al., 2008; Polezzi et al., 2010; San Martin et al., 2010; Zhou et al., 2010; Gu et al., 2011b; Kreussel et al., 2012; Luque et al., 2012). This method has the strength of increasing the signal-to-noise ratio in addition to allowing for better trial-by-trial measures. Nevertheless, it assumes equivalent baseline for each ERP component in different conditions. Given the overlap between P3 and FRN, this issue is particularly critical in outcome processing research. Indeed, the net amplitude of the FRN can be shifted to more positive values if the FRN for a particular condition is superimposed in a P3 that is particularly large. The second alternative method is to measure the base-to-peak difference for each deflection (e.g., defining the FRN as the difference between the most positive point and the most negative point in the 150–350 ms time window post-onset of feedback) (Holroyd et al., 2004a; Yeung and Sanfey, 2004; Frank et al., 2005; Toyomaki and Murohashi, 2005; Hajcak et al., 2006; Oliveira et al., 2007; Bellebaum et al., 2010; Chase et al., 2011). One problem with this measure is that it is especially susceptible to noise. Noise can be controlled by replacing the base of comparison with the mean amplitude in a time window around the base and by replacing the peak measure with the mean amplitude in a time window around the peak. A more fundamental problem is that the measure of the base (e.g., the beginning of the FRN) may be affected by the adjacent deflection (e.g., P2-like positivity).

Alternative methods that have been explored in recent years include isolating the activity associated with a particular ERP component using bandpass filtering (e.g., measuring the FRN after removing the slower frequency to which the P3 is associated) (Luu et al., 2003; Wu and Zhou, 2009; Gu et al., 2011a; Yu et al., 2011) or using temporospatial principal component analysis (PCA) (Carlson et al., 2011; Foti et al., 2011) or independent component analysis (ICA) (Gentsch et al., 2009). One limitation of these methods is that they strongly rely on decisions made by the researchers regarding the parameters used for bandpass filtering or during the identification of the PCA-derived or ICA-derived components that will be considered to represent the ERP components of interest. However, the emerging use of data-driven approaches for the selection of independent components (Wessel and Ullsperger, 2011) suggests that ICA might become a standard technique to decompose ERP components in the incoming years.

The problem of component overlap is inherent to all ERP research. In recent years, researches interested in how the brain processes outcomes have begun to consider ways to dissociate the contribution of the FRN and the P3 to the scalp-recorded ERP signal, and different methods have been proposed to achieve this goal. Given that the choice made among different measurement methods can have consequences, both in the empirical results that are found and in the conclusions that are proposed, future research should explore the strengths and limitations of different measurement methods and advance toward standard practices.

### Methodological challenge: employing multi-methods approaches (ERP/time-frequency, ERP/fMRI)

The temporal resolution of the EEG signal allows studying the neurocognitive mechanism of outcome processing and learning with a high level of temporal detail (milliseconds). The extraction of outcome-locked ERPs from the EEG signal is a good way to identify and study regularities in the neural processing of outcomes. However, ERP research presents two important limitations: it is relatively insensitive to the involvement of large-scale brain networks and it has a limited ability to identify the neural generators of the scalp-recorded signals (i.e., poor spatial resolution). Complementary techniques can be used to overcome such limitations. Specifically, time-frequency-based approaches could complement ERP studies by shedding light on the interactions among large-scale networks and fMRI can be used in a complementary fashion to help resolve the so-called inverse problem: a given distribution of scalp-recorded electrical activity could have been generated by any one of a large number of different sets of neural generators.

In order to provide a better account of the neural dynamics of outcome processing, EEG/ERP studies can benefit from the time-frequency information that is present in the same EEG signal from which ERPs are extracted. Event-related oscillations can be extracted using time-frequency decomposition analyses such as complex wavelet convolutions, from which one can obtain estimates of phase synchronization, spectral coherence, power-power correlations, spectral Granger causality, and cross-frequency coupling among recording sites (for a review see Cohen et al., 2011). Assessing large-scale networks is especially important to better understand the dynamics of feedback-guided learning, given that learning probably corresponds to changes in connectivity between neural populations (Hebb, 1949). Specifically, time-frequency-based approaches could be used to test hypotheses about inter-regional coupling between areas that probably interact during feedback-guided learning, such as medial prefrontal, sensory, and motor cortices.

A fundamental problem of EEG/ERP research is the inverse problem, by which a given distribution of scalp-recorded electrical activity could have been generated by any one of a large number of different sets of neural generators. Despite this problem, convergent evidence suggests that it is highly probable that the neural sources of the FRN are located in the mPFC (Miltner et al., 1997; Gehring and Willoughby, 2002; Ruchsow et al., 2002; Holroyd et al., 2004b; van Schie et al., 2004; Muller et al., 2005; Nieuwenhuis et al., 2005b; Hewig et al., 2007; Yu and Zhou, 2009; Yu et al., 2011). The picture is much less clear for the P3, for which neural sources are probably distributed over different regions of the cortex. In the same way that theories of the FRN have benefited from theories about the functional role of the mPFC (and vice versa), our understanding of the functional role of the P3 and its subcomponents during outcome processing could benefit from a more precise identification of its neural sources.

Increasingly, efforts are being made in order to use fMRI data to constrain the solution of the algorithms used for source localization analyses for ERPs. Although this approach does not completely solve the inverse problem, it increases the likelihood of identifying the actual sources of ERP activity. Especially promising is the use of joint ERP and fMRI ICA that have been used to reveal a number of cortical and subcortical areas involved in the generation of the response-locked ERN (Edwards et al., 2012).

### Empirical challenge: determining the impact of task demands

One of the primary goals of the brain is the adaptive control of behavior, but the exact definition of what constitutes an adaptive behavior may vary across situations, and different brain mechanisms can be recruited to guide behavior depending on the demands imposed by the task at hand. ERP studies of outcome processing have employed experimental paradigms whose behavioral demands range from the passive observation of monetary gains and losses in a computer screen (e.g., Yeung et al., 2005; Potts et al., 2006) to feedback-guided decision-making tasks requiring the inference of probabilistic rules governing state-outcome contingencies (e.g., Bellebaum and Daum, 2008; Chase et al., 2011; Walsh and Anderson, 2011). This breadth is a rich source of evidence, but at the same time is a likely factor underlying the difficulty for extracting generalizable conclusions about how outcome properties affect each ERP component.

The question of what ERP component better predicts behavioral adjustment is a good example of how different tasks may recruit different mechanisms to achieve the same overall goal (e.g., to accumulate monetary rewards). Behavioral adjustment might depend on the system underlying the FRN in tasks de-incentivizing the adoption of an explicit rule (cf. Cohen and Ranganath, 2007), and on the system underlying the P3 in tasks incentivizing the use of explicit probabilistic beliefs (cf. Chase et al., 2011). This possible dissociation has an interesting parallel with a distinction proposed by Daw et al. (2005) between model-free reinforcement learning (mediated by the basal ganglia in a way that is consistent with the RL-theory of the FRN) and model-based reinforcement learning (mediated by brain regions that have shown P3-like activity, such as the DLPFC, and MTL structures).

Future studies might elucidate whether different ERP components reflect the recruitment of different learning systems depending upon different tasks demands. In general, it is foreseeable that future ERP studies will, in greater proportion, be concerned with how different task demands and task contexts modulate the effect that outcomes have on ERP components.

### Conceptual challenge: understanding the temporal cascade of outcome processing in the brain

ERP studies of outcome processing have been focused primarily on the FRN and secondarily on the P3. There have been few attempts to present an account of outcome processing in the brain that integrates FRN and P3 effects. In this regard, the independent coding model (Yeung and Sanfey, 2004) is the dominant proposal to date. By proposing that the FRN codes valence but is insensitive to magnitude and that the P3 shows the opposite pattern, this model presents these two ERP components as measures reflecting brain processes that are completely independent from each other. Moreover, the temporal succession between the frontally distributed FRN and the parietally distributed P3 is not taken into account; for this model it does not matter if valence is evaluated before magnitude or if magnitude is evaluated before valence. Actually, the temporal cascade of ERP components is generally disregarded even in studies finding evidence that contradicts the independent coding model.

ERP effects are not always the real-time reflections of the underlying processes. For example, according to the LC-P3 hypothesis the P3 is an indirect index of LC phasic responses occurring 300–400 ms before the peak of the P3. However, the ubiquitous temporospatial succession between the frontally distributed FRN and the parietally distributed P3 probably reveals something meaningful about the manner in which the brain processes and learns from outcomes. Much of the contribution of ERP research to cognitive neuroscience has to do with describing the temporal cascade of neurocognitive processes involved in solving a particular task. ERP studies of outcome processing, in this regard, have traditionally sub-exploited the temporal resolution of the ERP technique.

There is also evidence suggesting that the FRN and the parietal P3 are not the only ERP deflections reflecting outcome processing in the brain. Studies could benefit from measuring the frontally distributed P3a that peaks 60–80 ms earlier than the P3b (Courchesne et al., 1975; Squires et al., 1975; Friedman et al., 2001), which according to a visual inspection is present in most of the ERP studies of outcome processing. These studies might also benefit from quantifying a positive deflection that typically began 150 ms after stimulus onset in frontal sites, and that according to Goyer et al. (2008) is modulated by outcome magnitude. This positivity, which in terms of latency could be referred to as “P2,” (see Figure 1A) probably represents an early stage of the slow-wave P3a on which the FRN is superimposed.

By considering the whole sequence of ERP deflections that are modulated by outcomes, ERP studies might contribute to build models of outcome processing that incorporate the inter-relationship between different cognitive processes that probably take part in outcome processing and learning, such as attention, valuation, and memory.

### Outlook

In order to control the behavior in an adaptive manner the brain has to learn how certain situations predict positive or negative outcomes and what actions are appropriate in a given situation. ERP research has shown that the brain is able to evaluate and learn from outcomes within a few hundred milliseconds of their occurrence. However, the accumulated literature presents a high degree of scientific uncertainty regarding the factors that modulate different ERP components during outcome processing. The FRN, in most cases, is larger for negative than for positive outcomes, but the effect of outcome magnitude and outcome probability over the FRN is less clear and contradicting evidence has been found regarding the relationship between the FRN and behavioral adjustment. The P3 is consistently more positive for large magnitude and unexpected outcomes than for small magnitude and expected outcomes, respectively, but the modulatory effect of feedback valence and the relationship between P3 and behavioral adjustment is much less clear.

During the last decade, ERP research has accumulated rich evidence of how outcomes are processed in the human brain. The next decade of research will probably be characterized by growing efforts to reach conclusions that generalize across task scenarios demands. In doing so, this research will advance our understanding of how the brain is able produce adaptive behavior in a large variety of situations.

### Conflict of interest statement

The author declares that the research was conducted in the absence of any commercial or financial relationships that could be construed as a potential conflict of interest.
